# Neuronal and Cerebrovascular Complications in Coronavirus Disease 2019

**DOI:** 10.3389/fphar.2020.570031

**Published:** 2020-11-20

**Authors:** Mudasir S. Andrabi, Shaida A. Andrabi

**Affiliations:** ^1^ Capstone College of Nursing, University of Alabama, Tuscaloosa, AL, United States; ^2^ Department of Pharmacology and Toxicology, University of Alabama at Birmingham School of Medicine, Birmingham, AL, United States; ^3^ Department of Neurology, University of Alabama at Birmingham School of Medicine, Birmingham, AL, United States

**Keywords:** severe acute respiratory syndrome coronavirus-2, coronavirus disease 2019, neurons, stroke, angiotensin-converting enzyme 2, axonal transport, neurodegeneration

## Abstract

Coronavirus disease 2019 (COVID-19) is a pandemic disease resulting from severe acute respiratory syndrome coronavirus-2 (SARS-CoV-2) infection, primarily in the respiratory tract. This pandemic disease has affected the entire world, and the pathobiology of this disease is not yet completely known. The Interactions of SARS-CoV-2 proteins with different cellular components in the host cell may be necessary for understanding the disease mechanism and identifying crucial pharmacological targets in COVID-19. Studies have suggested that the effect of SARS-CoV-2 on other organs, including the brain, maybe critical for understanding the pathobiology of COVID-19. Symptoms in COVID-19 patients, including impaired consciousness dizziness, headache, loss of taste and smell, vision problems, and neuromuscular pain, suggest that neuronal complications comprise a crucial component of COVID-19 pathobiology. A growing body of literature indicates that SARS-CoV-2 can enter the brain, leading to neuronal defects in COVID-19 patients. Other studies suggest that SARS-CoV-2 may aggravate neuronal complications due to its effects on the cerebrovascular system. Emerging pieces of evidence show that stroke can be one of the leading neurological complications in COVID-19. In this review, we describe the observations about neuronal complications of COVID-19 and how SARS-CoV-2 may invade the brain. We will also discuss the cerebrovascular problems and occurrence of stroke in COVID-19 patients. We will also present the observations and our views about the potential pharmacological strategies and targets in COVID-19. We hope this review will help comprehend the current knowledge of neuronal and cerebrovascular complications from SARS-CoV-2 infections and highlight the possible long-term consequences of SARS-CoV-2 on the human brain.

## Introduction

The severe acute respiratory syndrome coronavirus-2 (SARS-COV-2) has infected tens of millions, with more than one million deaths worldwide since first detected in early December 2019 in Wuhan, China (Holder and Reddy, 2020; Hu et al., 2020; Vallamkondu et al., 2020). This infection has resulted in the pandemic coronavirus disease 2019 (COVID-19), which is primarily considered a respiratory tract disease (Holder and Reddy, 2020; Hu et al., 2020). The SARS-CoV-2 is an exceptionally infectious type of coronavirus compared to previous coronaviruses known to infect humans (Vallamkondu et al., 2020). Prior epidemics due to coronaviruses include the SARS-CoV infections that resulted in thousands of death in 2003 in China due to acute respiratory distress (Drosten et al., 2003; Vallamkondu et al., 2020) and the Middle East Respiratory Syndrome coronavirus (MERS-CoV) infections, which also caused more than a thousand deaths in the Middle East in 2012 (Zaki et al., 2012). SARS-CoV-2 is unique because it is most contagious and can survive on different surfaces for long periods. The number of SARS-CoV-2-positive infections and COVID-19 deaths continues to increase dangerously. COVID-19 was first recognized primarily as respiratory tract infection progressing into low to severe grade pneumonia within days after infection in the patients (Vallamkondu et al., 2020). The general symptoms of COVID-19 include fatigue, fever, chills, muscle pain, chills, headache, sore throat, cough, loss of smell and taste, and sometimes hallucinations and dizziness. Severe symptoms include stern chest pain, trouble in breathing, confusion, and reasoning inability. The severely affected patients require immediate hospitalization and may need a mechanical ventilator to support respiration. Sudden cardio-respiratory failure is the leading cause of death in COVID-19 patients.

The precise pathobiology of the COVID-19 is not clear, and yet there is no drug or a vaccine available to stop this pandemic disease. Additionally, it is not clear whether the development of the COVID-19 is solely due to localized respiratory tract infection or the disease involves other vital organs. A vast number of SARS-CoV-2 positive patients are asymptomatic. However, cardiorespiratory arrest is a prevalent cause of mortality in patients that are dying of COVID-19. It is thought that altered immune and inflammatory responses in the respiratory tract play a substantial role in the pathobiology of COVID-19 (Manjili et al., 2020; Merad and Martin, 2020), it is also likely the interaction of different viral proteins with vital cellular processes may contribute to the pathobiology of the COVID-19 (Gordon et al., 2020; Holder and Reddy, 2020). Recent studies focused on mapping the interactions of SARS-CoV-2 with human proteins have indicated that alterations in different cellular processes may be involved in the pathobiology of COVID-19 (Gordon et al., 2020). There is a growing consensus that COVID-19 may also involve the brain, and the effects of the SARS-CoV-2 on the neurons in different brain areas may likely contribute to the pathobiology of this disease or sudden death due to cardio-respiratory arrest if the virus infects the cardio-respiratory centers in the brain stem (Gordon et al., 2020; Mao and Jin, 2020). Besides, cerebrovascular endothelial cells is another likely target of SARS-CoV-2 to induce some aspects of COVID-19 pathobiology (Zubair et al., 2020). A developing body of literature indicates that stroke can be one of the primary neurological complications in COVID-19 patients (Zayet et al., 2020; Zubair et al., 2020).

## Neurological Manifestations in Coronavirus Disease 2019

Various studies have shown manifestations of neurological symptoms in COVID-19 patients. Data collected from January 16, 2020, to February 19, 2020, in a retrospective study involving 214 consecutively hospitalized COVID-19 patients in Wuhan, China showed evidence of neurological manifestations with SARS-CoV-2 infection (Mao et al., 2020). This study found that around 36% of COVID-19 patients displayed a wide range of neurological symptoms, including headache, dizziness, acute cerebrovascular disease, ataxia, seizure, loss of taste, vision problems, neuromuscular pain, and impaired consciousness. Patients with more severe infection showed more severe neurological symptoms (Mao et al., 2020). A case study on a COVID-19 patient found that in addition to fever and respiratory symptoms, the patient also developed neurological symptoms, including myalgia and altered consciousness (Yin et al., 2020). Another case study involving a female COVID-19 patient showed the clinical manifestations of cough, fever and altered mental status. The MRI brain scans of this patient showed hemorrhagic lesions resembling acute necrotizing hemorrhagic encephalopathy (Poyiadji et al., 2020). Several reports indicate severe neurological problems, including the pervasiveness of encephalitis and stroke in COVID-19 patients (Barrios-Lopez et al., 2020; Bernard-Valnet et al., 2020; Pilotto et al., 2020). In another retrospective study on COVID-19 patients admitted to ASST Papa Giovanni XXIII, Bergamo, Italy, further confirmed that neurological manifestations are common observations seen with SARS-CoV-2 infection. Out of 1760 COVID-19 patients in this study, 137 displayed neurological manifestations that ranged from cerebrovascular issues to peripheral and central nervous system problems (Rifino et al., 2020). In most patients, the neurological manifestations were observed after the resolution of COVID-19 symptoms (Rifino et al., 2020), suggesting that some COVID-19 patients are likely to develop neurological issues in the long run.

SARS CoV-2 and SARS-CoV are closely related coronaviruses, and both these viruses likely share some common pathobiological features. Studies on SARS-CoV have also provided compelling pieces of evidence that coronaviruses can infect the brain (Xu et al., 2005). SARS-CoV like particles were identified as the brain autopsy samples of a SARS-CoV infected patient. The brain showed the presence of necrotic lesions with infiltrating monocytes, macrophages, and lymphocytes, indicating activation of immunological responses as the virus invades the brain. The neuronal symptoms started when the condition of the patient was normal with some resolving pneumonia, suggesting that the neuronal manifestation are independent of pulmonary problems and it is not that hypoxia-like condition in the brain due to ailing lungs may lead to neuronal problems in SARS patients (Xu et al., 2005). In another case, involving a 32-year-old female patient infected with SARS CoV displayed generalized convulsions and the presence of SARS-CoV viral particles and signs of CNS infection (Lau et al., 2004). Edema and neuronal degeneration and the presence of SARS CoV particles or the viral genomic sequences together with circulating immune cells in different brain parts were identified in SARS-CoV infected patients (Gu et al., 2005). Several studies related the SARS CoV infection in patients with late-stage SARS with neuronal and psychologic abnormalities (Chan et al., 2006; Yip et al., 2010). Similar neurological observations, including encephalitis, infectious and toxic neuropathies, and Guillain-Barré syndrome, were found in coronavirus MERS-CoV infected patients (Kim et al., 2017). Several studies in the animal models complemented these case studies. These studies indicate that the effect of SARS-CoV-2 on the brain may be a critical part of the COVID-19 pathobiology, and neurological manifestations should be carefully monitored in COVID-19 patients. Activation of systemic inflammatory responses via an acute increase in pro-inflammatory cytokines, including interleukin-6 (IL-6) termed as Cytokine Storm, has been linked to disease severity and death in COVID-19 patients (Hashizume, 2020; Hojyo et al., 2020; Lukan, 2020). IL-6 like pro-inflammatory interleukins are also elevated in some neurodegenerative diseases such as Parkinson’s disease (PD) (Kwiatek-Majkusiak et al., 2020), suggesting that patients with neurodegenerative diseases may be more susceptible to the severity of COVID-19. Although the relationship between COVID-19 severity and the progression of neurodegeneration diseases is not yet established, studies have shown that the COVID-19-related mortality rate is significantly higher in PD patients (Fasano et al., 2020; Zhang et al., 2020). A study involving two PD patients indicates that early diagnosis of COVID-19 may be challenging as COVID-19 may mimic some PD symptoms (Hainque and Grabli, 2020). This study emphasized and highlighted the importance of COVID-19 severity in PD patients (Hainque and Grabli, 2020). COVID-19 might also affect α-synuclein clearance, resulting in exacerbated α-synuclein aggregation in PD (Pavel et al., 2020). Therefore, COVID-19 patients with PD and other neurodegenerative diseases must be carefully observed for long-time even after the recovery from COVID-19.

It is not clear how SARS-CoV-2 can infect the brain, as the binding to a host cell receptor is a critical step for the entry of a virus into a host cell. The spike (S) protein on the coronaviruses plays an essential role in recognizing and binding different host-cell receptors. Studies have shown that SARS-CoV can utilize angiotensin-converting enzyme 2 (ACE2) as the receptor to move into a host cell and engages TMPRSS2 protease for the priming of the S-protein (Li et al., 2007). MERS-CoV and PRCV use dipeptidyl peptidase 4 (DPP4) while other coronaviruses like HCoV-NL63, TGEV recognize aminopeptidase N (APN) as host cell receptors (Li, 2015). The sequence similarities between SARS-CoV and SARS-CoV-2 and interaction mapping of SARS-CoV-2 proteins with the human proteome indicate that the S-protein of the virus also uses ACE2 and TMPRSS2 to enter the host cells (Baig et al., 2020; Hoffmann et al., 2020; Ju et al., 2020; Shi et al., 2020; Yan et al., 2020). Protein expression studies have shown that respiratory tract epithelial cells express high levels of ACE2 (Jia et al., 2005), and it is one of the reasons why the respiratory tract is the primary site of SARS-CoV or SARS-CoV2 infections. However, ACE2 may not be the sole receptor for host-cell viral infection by SARS-CoV/SARS-CoV-2, or these viruses may use other modes of entry that are not yet identified. This notion is supported by the observation that cells with very low or undetectable levels of ACE2 protein expression, including neuronal cells in the brain or hepatocytes, are also infected. In contrast, some cells, including cells lining the human gut or the endothelial, are not readily infected despite expressing high levels of ACE2 (Li et al., 2020). In addition, recent studies shown that human lungs have the lowest levels of ACE2 mRNA, where it is highest in the gut (Xu et al., 2020). Therefore, it is crucial to explore all the possible options that all types of coronaviruses may use to enter the host cells in order to develop therapeutic drugs targeting viral/host cell interaction for SARS-CoV-2. One possibility for the entry of SARS-CoV/SARS-CoV-2 into the brain is via blood or other body fluids, including lymph. However, an autopsy of SARS-CoV patients showed SARS-CoV in the brain samples but not in the in parenchymal cells with no pathogenic changes in several other organs and tissues including the pancreas, the adrenal gland, the heat, the thyroid gland, and the skeletal muscle. These studies indicate the blood or lymph route may not be the route of entry for coronaviruses into the brain. In addition, studies on MERS-CoV using mice showed that low inoculum doses of the virus only infect the brain (Li et al., 2016). Other studies have shown that the coronavirus can travel to the brain via axonal transport from the olfactory tract (Butowt and Bilinska, 2020). Once in the brain, the SARS-CoV-2 can infect large neuronal populations in different brain areas, including the neurons in the brain stem, which can be extremely dangerous leading to respiratory failure and sudden death besides other neurological complications, including encephalopathy. The presence of immune cells, together with viral particles, also suggests that the infiltration of virus-containing non-resident immune cells into the brain may be not be ruled out as a route of SARS-CoV-2 infection in the brain in addition to axonal transport via peripheral nerves. BBB disruption by SARS-CoV-2 protein components could also be a route of entry into the brain but studies are required to precisely identify whether the disruption of BBB may also facilitate the neuro-invasion of SARS-CoV-2.

## Cerebrovascular Complications and Stroke in Coronavirus Disease 2019

Stroke may be another complication in COVID-19 patients. A study on six stroke patients of different ages confirmed that all these cases were COVID-19 positive. However, this study was not sufficient to conclude that there is a relationship between SARS-CoV-2 and the occurrence of stroke as the majority of these patients had underlying vascular risk factors, including hypertension and cardiovascular problems. Nevertheless, this study indicates that COVID-19 may be associated with stroke (Beyrouti et al., 2020). Likewise, in another study, patients younger than 50 years of age admitted to a hospital in New York with acute large-vessel stroke tested all positive for SARS-CoV-2 (Beyrouti et al., 2020). Stroke was also observed in a significant number of COVID-19 patients in Wuhan China (Mao et al., 2020). A study conducted on COVID-19 patients in Bergamo, Italy, showed that out of 137 patients with neurological symptoms, 38.7% developed cerebrovascular diseases that include ischemic/hemorrhagic strokes, transient ischemic attacks, and cerebral venous thrombosis (Rifino et al., 2020). As with the other neurological manifestations, these cerebrovascular diseases displayed after the COVID-19 symptoms were resolved in most patients (Rifino et al., 2020). Although pro-thrombotic condition leading thromboembolism and elevated D-dimer levels are a probable cause of strokes in SARS-CoV-2 infection, it is essential to consider that the effect of SARS-CoV-2 on cerebrovascular endothelium may be a cause of stroke in COVID-19 patients and complicate the COVID-19 neurological manifestations.

While ACE and ACE2 are both involved in renin-angiotensin pathways, but their actions counterbalance each other’s effects (Paul et al., 2006; Luhtala et al., 2009). ACE converts angiotensin-I into angiotensin-II whereas ACE2 catalyzes the formation of angiotensin-(1–7) from angiotensin II and angiotensin-(1–9) from angiotensin-I, which can be further catalyzed to angiotensin-(1–7) by ACE (Paul et al., 2006). The traditional view is that angiotensin-II formed by ACE from angiotensin-I has vasoconstriction effect while angiotensin-(1–7) leads to vasodilation effects (Paul et al., 2006; Luhtala et al., 2009). It is likely that the binding of SARS-CoV-2 to ACE2 sequesters this enzyme from performing its activity in the renin-angiotensin pathway. The consequent effects may lead to more vasoconstriction and clot formation resulting in poor blood flow to different organs, including the brain. Supporting this view, a study showed that anticoagulant therapy using low molecular weight heparin resulted in better prognosis in COVID‐19 patients who had high circulating D-dimer levels (Tang et al., 2020). Likewise, thrombolytic therapy using Tissue Plasminogen Activator (tPA) improved the condition of the patients and prevented the requirement of mechanical ventilators by improving the oxygen requirements of COVID-19 patients (Christie et al., 2020).

Moreover, the effect of viral proteins on different physiological functions in endothelial cells lining the blood vessels may further provoke vascular dysfunction, including clot formation. Therefore, pharmacological strategies focused on vasodilation or anti-coagulation may be effective in reducing the cerebrovascular problems in COVID-19 patients. Besides, the identification of molecular mechanisms of how different viral proteins affect cellar function upon interaction with several crucial host cell proteins in cerebrovascular endothelial cells (Gordon et al., 2020) may be very critical for developing pharmacological strategies for COVID-19 and related cerebrovascular complications including stroke. The cytokine storm in COVID-19 results in IL-6 dependent alterations in the expression of vascular endothelial growth factor and E-cadherin expression on endothelial cells contributing to vascular leakage and pulmonary hypertension, and respiratory distress in COVID-19 patients (Lukan, 2020; Moore and June, 2020). Whether IL-6 causes similar leakage in the cerebral vasculature, resulting in brain endothelial cell damage, is unknown. SARS-CoV-2 envelope protein (E) can interact with tight junction-associated protein PALS1 resulting in severe damage to the epithelial barrier (Teoh et al., 2010). The cerebral endothelial tight junctions serve as a critical component of BBB; it is intriguing to see whether SARS-CoV-2 components interfere with the tight junction proteins of brain endothelial cells, thereby altering the integrity of the BBB. A study showed that components of SARS-CoV-2 spike protein could damage BBB and endothelial cells by initiating a pro-inflammatory response via an increase in matrix metalloproteinases (Buzhdygan et al., 2020). Alterations in BBB and endothelial cells can complicate the brain damage in stroke and cerebrovascular diseases (Venkat et al., 2017; Haruwaka et al., 2019). It is plausible that the COVID-19 prognosis may be worse in individuals with cerebrovascular disorders or those who are susceptible to develop cerebrovascular diseases, and the outcome may likely be worse if a stroke patient develops COVID-19.

## Conclusion

As a significant number of the COVID-19 patients develop neurological manifestations and stroke, the axonal transport of SARS-CoV-2 via peripheral nerves likely allows invasion of this virus into the brain, and SARS-CoV-2 viral particles may directly affect the cerebrovascular endothelial cells to impede the blood flow to some regions of the brain resulting in ischemic or hemorrhagic strokes. In the brain, SARS-CoV-2 can alter the functions of neurons in different brain areas, including the cardiorespiratory centers in the brainstem. The effect of SARS-CoV-2 in the brain may complicate the pathology of COVID-19 and may precipitate in a variety of long-term neurological diseases. Death due to sudden respiratory arrest in some COVID-19 patients may not be ruled out due to the potential effects of SARS-CoV-2 on the neurons of the respiratory center in the brain stem. Therefore, neurological and cerebrovascular symptoms in COVID-19 patients must be taken very seriously, and COVID-19 patients should be monitored for any neurological complications and signs of a stroke for long time even after their recovery from the disease.

## Author Contributions

MA and SA planned, reviewed the literature, and wrote the manuscript.

## Conflict of Interest

The authors declare that the research was conducted in the absence of any commercial or financial relationships that could be construed as a potential conflict of interest.
